# Analysis of students' positive emotions around the green space in the university campus during the COVID-19 pandemic in China

**DOI:** 10.3389/fpubh.2022.888295

**Published:** 2022-08-09

**Authors:** Shaobo Liu, Yifeng Ji, Jiang Li, You Peng, Zhitao Li, Wenbo Lai, Tao Feng

**Affiliations:** ^1^The Department of Environmental Design, School of Architecture and Art, Central South University, Changsha, China; ^2^Urban Planning and Transportation Research Group, Department of the Built Environment, Eindhoven University of Technology, Eindhoven, Netherlands; ^3^Smart Transport Key Laboratory of Hunan Province, School of Traffic and Transportation Engineering, Central South University, Changsha, China; ^4^Department of Landscape Architecture, School of Architecture, South China University of Technology, Guangzhou, China; ^5^Urban and Data Science Lab, Graduate School of Advanced Science and Engineering, Hiroshima University, Higashi Hiroshima, Japan

**Keywords:** COVID-19, campus green spaces, positive emotion, perceived naturalness, place attachment, landscape preference, structural equation modeling

## Abstract

Green space around the university campus is of paramount importance for emotional and psychological restorations in students. Positive emotions in students can be aroused when immersed in green space and naturalness. However, to what extent can perceived naturalness influence students' positive emotion remains unclear, especially in the context of COVID-19 countermeasures. This study, therefore, attempts to investigate in-depth the nature and strength of the relationships between students' positive emotion and their perceived naturalness, place attachment, and landscape preference, which are potentially varying across universities in different social and environmental contexts and different restrictions policies regarding the COVID-19 pandemic. A course of questionnaire-based surveys was administered on two university campuses in Heilongjiang and Hunan Provinces, China, resulting in 474 effective samples. Structural equation modeling was used to explore the hypothetical conceptual framework of latent variables and the indicators. The findings indicate that the higher students' perceived naturalness results in greater positive emotion. Students' perceived naturalness in green spaces of campus has a positive effect on their place attachment and landscape preference. Moreover, the difference between mediate effects of place attachment and landscape preference were addressed, which verifies the contextual influences.

## Introduction

As a public health issue, the global pandemic of COVID-19 poses a serious threat to the built environment and human health (1). Many countries have therefore adopted lockdown to restrict people's activities to varying degrees (2, 3). In China, university students successively returned to school in the fall of 2020. Still, the outdoor activities of students were limited in most universities in order to prevent the potential spread of the epidemic (4). Under this circumstance, some university students experienced a reduction in social activities and encountered other troubles, such as financial stresses and academic frustrations, which may cause negative outcomes regarding emotional and mental health (5). Recent research addressed that approximately 45% of Chinese students had mental health problems during the COVID-19 period (6). In fact, even before the COVID-19 period, university students' negative emotions and mental health problems were commonplace due to time pressure, competition, and the pressure to achieve good academic grades (7). Several studies showed that individuals who exhibit a high level of negative emotion often show more distress, anxiety, and dissatisfaction (8, 9). Conversely, positive emotion is thought to alleviate psychological disorders such as depression and anxiety for university students (10, 11). Hence, there is an urgent need to effectively employ preventive measures to help students with emotional regulation (12, 13).

The psycho-evolutionary theory suggests that people's emotions can be positively affected by observing the natural environment (14). As the main place of contact with nature for university students, the campus green spaces (CGSs) include lawns, woods, and other landscaped spaces available for students to meet their emotional and psychological needs (15–17). van den Bogerd et al. (18) summarized the restorative effects of the CGSs on university students' emotions, including “reducing harm,” “restoring capacities,” and “building capacities” (18), all of which involve students' perceived naturalness of the CGSs. A significant positive correlation between students' perceived naturalness and their restoration and health was evidenced (17), which indicates the effect of perceived naturalness in regulating students' emotions in the CGSs.

However, although green spaces have similar characteristics to a certain extent, it is not reasonable to regard the perceived naturalness of the CGSs in different areas as the same as students' restorations (19). Conceptually, the perceived naturalness in different regions is localized to cultural, social, and environmental contexts (20), involving place attachment and landscape preference (21, 22). Place attachment is defined as a complex emotional or psychological bond between an individual and the environment (23). Previous studies claim that features of the natural environment affect individuals' attachment to place (24). An increase in place attachment is thought to improve emotions and promote psychological recovery (25). It can be inferred that, when attachment decreases, emotional improvement and psychological recovery can also be disrupted. Moreover, place attachment includes an individual's sense of belonging and familiarity with the place, which is related to landscape preference (24). The degree to which people are attached to the natural environment can affect their degree of preference and emotional state (26). Students may have different degrees of place attachment and landscape preference for the CGSs in different regions, which may lead to differences in the mechanisms by which perceived naturalness affects students' restorations.

At present, university students from different regions of China have experienced different periods of lockdown. The perception of naturalness, emotions, and the level of stress are affected by the regulations depending on the varying intensity of COVID-19 spreads (27). Researchers confirmed that individuals who reported feeling nature deprived during the COVID-19 lockdown experienced more emotional swings and mental health problems (28). More importantly, the lockdown associated with COVID-19 provided a new way for individuals to perceive the value of green space (29). Their attachment to and preference for nature has been amplified, which may also have implications for the relationship between perceived naturalness and emotions. Thus, comparative research in different social and environmental contexts will help broaden our understanding of the relationships between perceived naturalness and emotions and the mental health of university students. However, few published studies compared the different effects of perceived naturalness on emotions across different regions. Studies have not yet explored the potential effects of perceived naturalness of the CGSs on university students' emotions and the related mediate effects of place attachment and landscape preference in the context of the COVID-19 pandemic.

This study aimed to explore the hypothetical effects of place attachment and landscape preference in the relationship between the perceived naturalness of CGSs and university students' positive emotions under the restriction policies and regulations to control the spread of the COVID-19 pandemic. We comparatively investigated the hypotheses considering the social and environmental differences between two universities in Hunan and Heilongjiang provinces, China. Due to the different epidemiological conditions during the COVID-19 period in China, these two universities imposed control regulations with different intensities. The findings are expected to provide up-to-date contextual insights into the relationship between perceived naturalness and positive emotion and to systematically understand the mechanism that perceived naturalness in CGSs evokes the student's positive emotion. Furthermore, the implications are expected to help the planning and management of university green spaces for improving the mental health and well-being of university students.

## Conceptual framework

### The effect of perceived naturalness and emotions

Perceived naturalness refers to the proximity of the landscape to the perceived natural state (30), while emotion is defined as a value judgment that relates external events to inner concerns (31). A wealth of evidence has been provided for verifying the relationship between the perceived naturalness of the CGSs and university students' emotions (17, 18). Many natural elements, such as abundant plant species and large areas of vegetation may help students improve their emotional state and mental health in their daily lives (32). For instance, Malekinezhad et al. (33) found that university students' perceptions of the campus with green qualities may enhance positive emotion and contribute to improving mental health (33). On the other hand, a lack of natural elements in the environment can lead to an increase in negative emotions such as anxiety, impulsiveness, and sadness among university students (34).

Literature studies on the COVID-19 pandemic revealed that natural deprivation associated with lockdown leads to restriction of physical activity and experience of negative emotions such as anxiety and depression among university students (27, 35). On the contrary, exposure to the natural environment during COVID-19 helps enhance an individual's perception of nature and reduces negative emotions such as anger, fear, and confusion (36). Despite the rapid control of COVID-19 in China in 2020, sporadic outbreaks and lockdowns are still occurring (37). Therefore, exploring how the perceived naturalness of the CGSs can affect university students' positive emotions during the COVID-19 pandemic. Due to differences in epidemic status and lockdown regulations, the relationship between the perceived naturalness of the CGSs and university students' positive emotions needs to be understood across social and environmental contexts (19, 38). Thus, this study proposed that hypothetical regional differences exist in the influences of the university students' perceived naturalness of CGSs on their positive emotions across different universities characterized by social and environmental features.

### The mediate effect of place attachment

Place attachment, as place identification and place dependence, is a positive emotional connection established between people and places through memory and exposure to the environment (39, 40) is and associated with perceived naturalness (41). Higher perceived naturalness results in stronger attachment, more exposure, and experience with the added benefits of contact with nature (16, 17). For university students, natural environments are more popular than urban environments (42). The students' perceived naturalness of CGSs can lead to the development of place attachment (43). In contrast, the lack of natural elements around the campus causes the absence of students' perceived naturalness and place attachment (44).

As an emotional bond between people and important places, students' place attachment is linked to their emotions (44). Students' self-esteem and sense of belonging can be enhanced by place attachment, and their positive emotions, such as relaxation and happiness, can also be increased (45). Furthermore, place attachment is used to connect the perceived naturalness of CGSs and positive emotion (46). When students are frustrated, fearful, and stressed by external events, the CGSs are conducive in forming students' place attachment and evoking their positive emotions (47). However, the mediate effect of place attachment is unclear in the relationship between students' perceived naturalness and positive emotion during the COVID-19 pandemic.

### The mediate effect of landscape preference

Landscape preference is formed through the interaction between people and the natural environment (48). The environmental preference matrix summarized human preferences for landscape as “understanding” (coherence and legibility) and “exploration” (complexity and mystery) (49). Several pieces of evidence have been presented, which explain the relationship between landscape preferences and the perceived naturalness of the CGSs (50). Compared to artificial landscapes, university students have more preference for natural landscapes with lots of trees, open areas, and water (51, 52). The well-designed CGSs as high-quality landscapes can provide university students opportunities to reduce stress and increase social interactions (53).

CGSs provided natural spaces to meet university students' landscape preferences, which contributed to generating positive emotions among students in the university (54, 55). Moreover, university students exposed to a preferred environment tend to have more positive emotions and lower negative emotions (51). It also has been demonstrated that, as a basis for the restorative effects of the environment, preferences moderate the effect of perceptions of green space on positive emotion (56). After experiencing the COVID-19 lockdown, university students were more eager to be in outdoor environments (35, 57). The positive attitude toward being outside may have an impact on students' perceived naturalness and landscape preference, which may affect their positive emotions about enjoying the CGSs.

### Hypothetical structure

Several studies showed that social and cultural contexts influence the form and function of green spaces, which affects individuals' perceptions of the environment (38, 58). In this study, the mechanism of students' positive emotions influenced by the perceived naturalness of CGSs is assumed to vary depending on regional contexts. Moreover, this study aimed to examine the role of place attachment and landscape preference in the relationship between students' perceived naturalness of the CGSs and their positive emotions. It is assumed that the university students' positive emotion in the CGSs is proportional to their place attachment and landscape preference. In addition, the mediate effects of students' place attachment and landscape preference are hypothesized to link the perceived naturalness and positive emotion.

**Figure 1:**
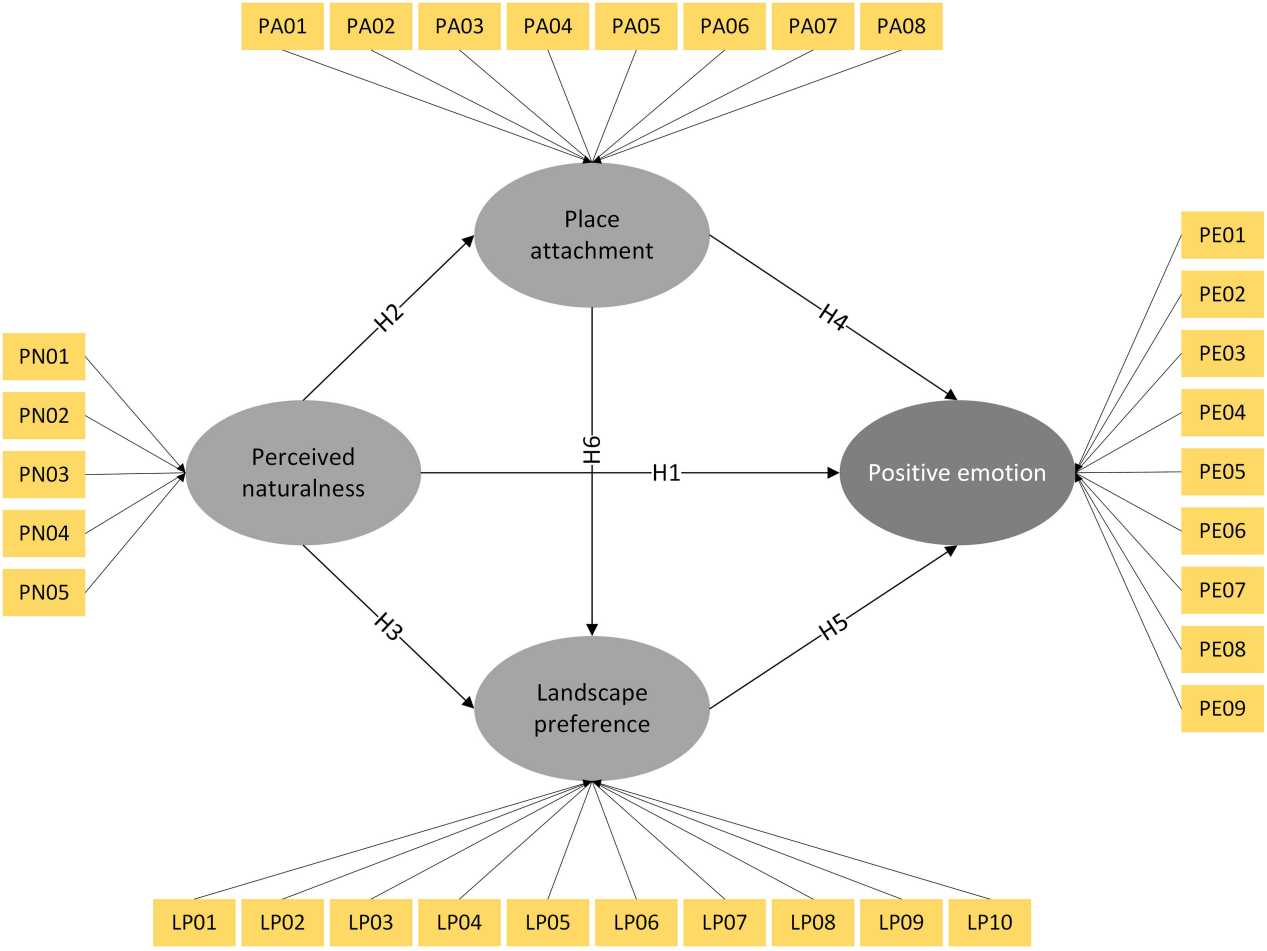
The diagram of the conceptual framework.

Figure 1 depicts the hypothetical relationships between the latent variables in the conceptual framework. We assumed that the students' perceived naturalness directly affects their positive emotion (H1), place attachment (H2), and landscape preference (H3). Place attachment and landscape preference play mediating roles to link perceived naturalness and positive emotion, respectively (H4 and H5). In addition, place attachment influences landscape preference (H6).

The exogenous variables used for constructing latent variables are listed in Table 1. The perceived naturalness was evaluated by the perception of natural attributes and natural feeling (17). Natural attributes refer to the flora, fauna, and sounds in the environment and are measured by PN01 and PN02. Natural feeling refers to the feeling brought by the natural environment and is measured through PN03 to PN05. Students' positive emotions were measured by the Positive Affect Scale, which constitutes the emotional component of subjective well-being (59). In the Chinese cultural context, positive emotion could be assessed through nine different emotions, (60) which were presented as PE01 to PE09. Place dependence and place identity are the two main dimensions of place attachment (45). In this study, place dependence, referring to the value of the place largely above other places, was measured by PA01 to PA04, and place identity, referring to the place as a part of oneself, is measured by PA05 to PA08. According to the environmental preference matrix (49), landscape preference was evaluated by four dimensions, including (1) coherence measured by LP01 and LP02, (2) legibility measured by LP03 to LP05, (3) complexity measured by LP06 and LP07, and (4) mystery measured by LP08 to LP10.

**Table 1:** Latent variables and the corresponding exogenous variables.

**Construct**	**Code**	**Measuring questions (Indictors)**
Perceived naturalness (PN)	PN01	I think the campus green spaces have many wild plants and animals.
	PN02	I can feel the strong natural voice in the campus green spaces.
	PN03	The campus green spaces make me feel friendly.
	PN04	I feel safe and calm in the campus green spaces.
	PN05	I feel the campus green spaces are wild.
Positive emotion (PE)	PE01	I feel active.
	PE02	I feel enthusiastic.
	PE03	I feel cheerful.
	PE04	I feel joyful.
	PE05	I feel excited.
	PE06	I feel proud.
	PE07	I feel inspired.
	PE08	I feel strong.
	PE09	I feel grateful.
Place attachment (PA)	PA01	The campus green spaces are comfortable and allow me to do the things I want.
	PA02	There is no other place like the campus green spaces.
	PA03	I can get more satisfaction in the campus green spaces than in other places.
	PA04	What I do on the campus green spaces is more important than what I do elsewhere.
	PA05	The campus green spaces allow me to see what I am interested in.
	PA06	I feel that the campus green spaces are part of my life.
	PA07	I have a strong identification with the campus green spaces.
	PA08	The campus green spaces are special, and I have good feelings about them.
Landscape preference (LP)	LP01	I think the various parts of the campus green spaces form a whole.
	LP02	The various parts of the campus green spaces form a beautiful landscape.
	LP03	I think the campus green spaces contain a multitude of elements and features.
	LP04	I think there are many intricate elements in the campus green spaces.
	LP05	I think the campus green spaces contain many functions.
	LP06	I can clearly understand the campus green spaces.
	LP07	I think the campus green spaces have clear markers.
	LP08	The campus green spaces make me want to investigate more.
	LP09	The campus green spaces are circuitous and intrigue me.
	LP10	The campus green spaces are far-reaching and mysterious.

## Methodology

### Study locations

**Figure 2:**
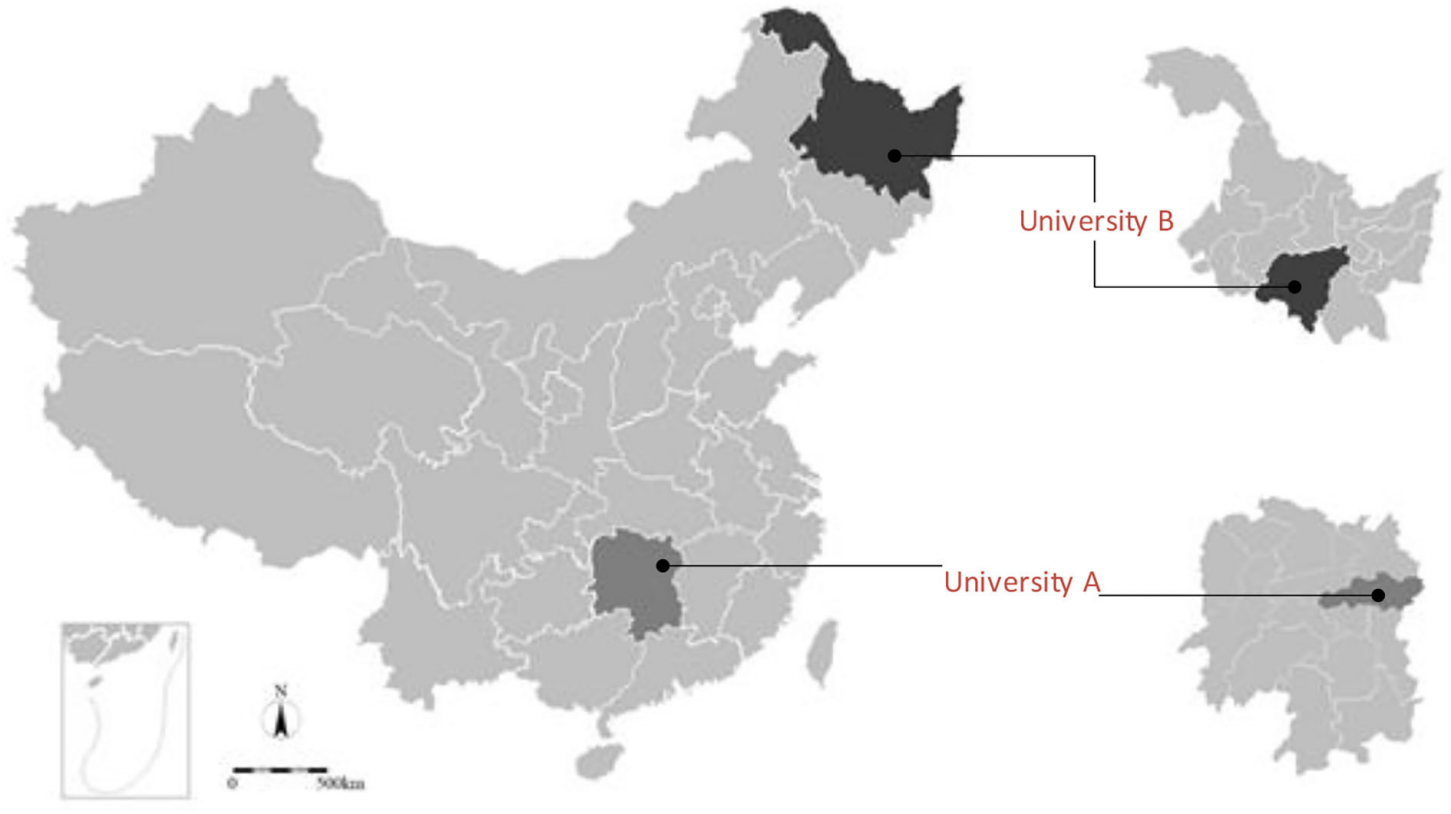
Locations of university campuses in different provinces in China.

The field surveys were conducted on two campuses, namely campus of university A (UA) in Changsha of Hunan Province and the campus of university B (UB) in Harbin of Heilongjiang Province, in China. According to the Köppen climate classification, Changsha is classified as Cfa and characterized by mild temperate climate and fully humid climate with hot summers, while Harbin is classified as Dwa and characterized by snow, dry winters, and hot summers (61). The locations of the studied universities are shown in Figure 2. UA started to reopen in September 2020 when the outdoor activity limits for COVID-19 pandemic control were phased out. Due to sporadic outbreaks in Heilongjiang Province at that time, UB implemented a stricter lockdown after students returned for the fall semester compared to UA. The characteristics of the two universities are listed in Table 2.

**Table 2:** Characteristics of the universities in this study.

	**UA**	**UB**
Location	Changsha, Hunan Province	Harbin, Heilongjiang Province
Number of students	Approx. 59000	Approx. 26000
The overall area	317 ha	136 ha

**Figure 3:**
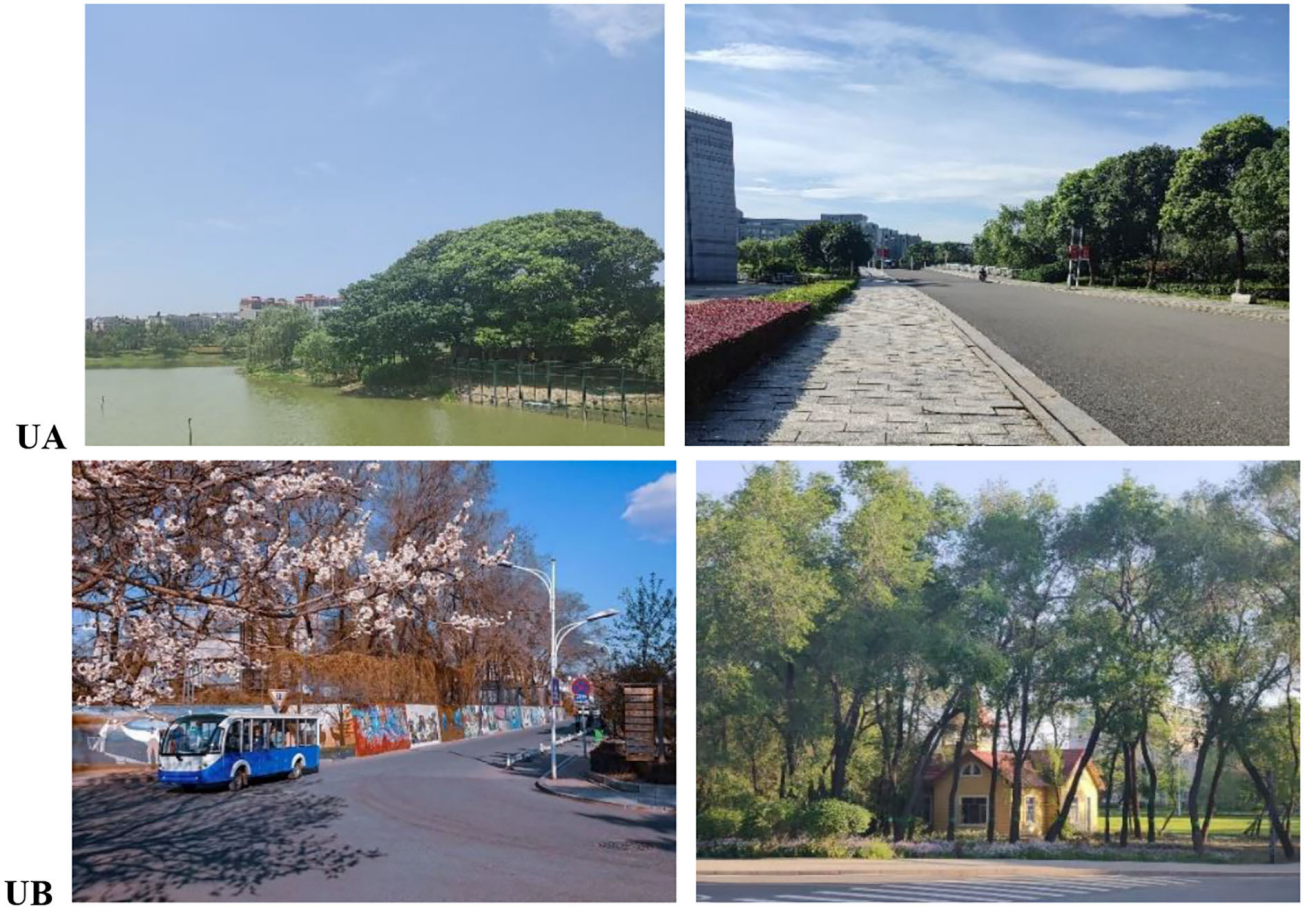
Scenes of the studied university campuses.

On the campus of UA, the green space is scattered and includes lawns, trees, and a river. There are many geese in the river. The outdoor activity area is crowded. Minor noises are caused by the green space under construction. Each green space has a unique character. The green areas in UB consist of large lawns, distinct flora, and fauna. A rich variety of plants is observed. The river flows through the campus. There are good opportunities for outdoor activities, good natural quality, and strong attraction. The scenes of the two campuses are shown in Figure 3.

### Measures and survey

**Figure 4:**
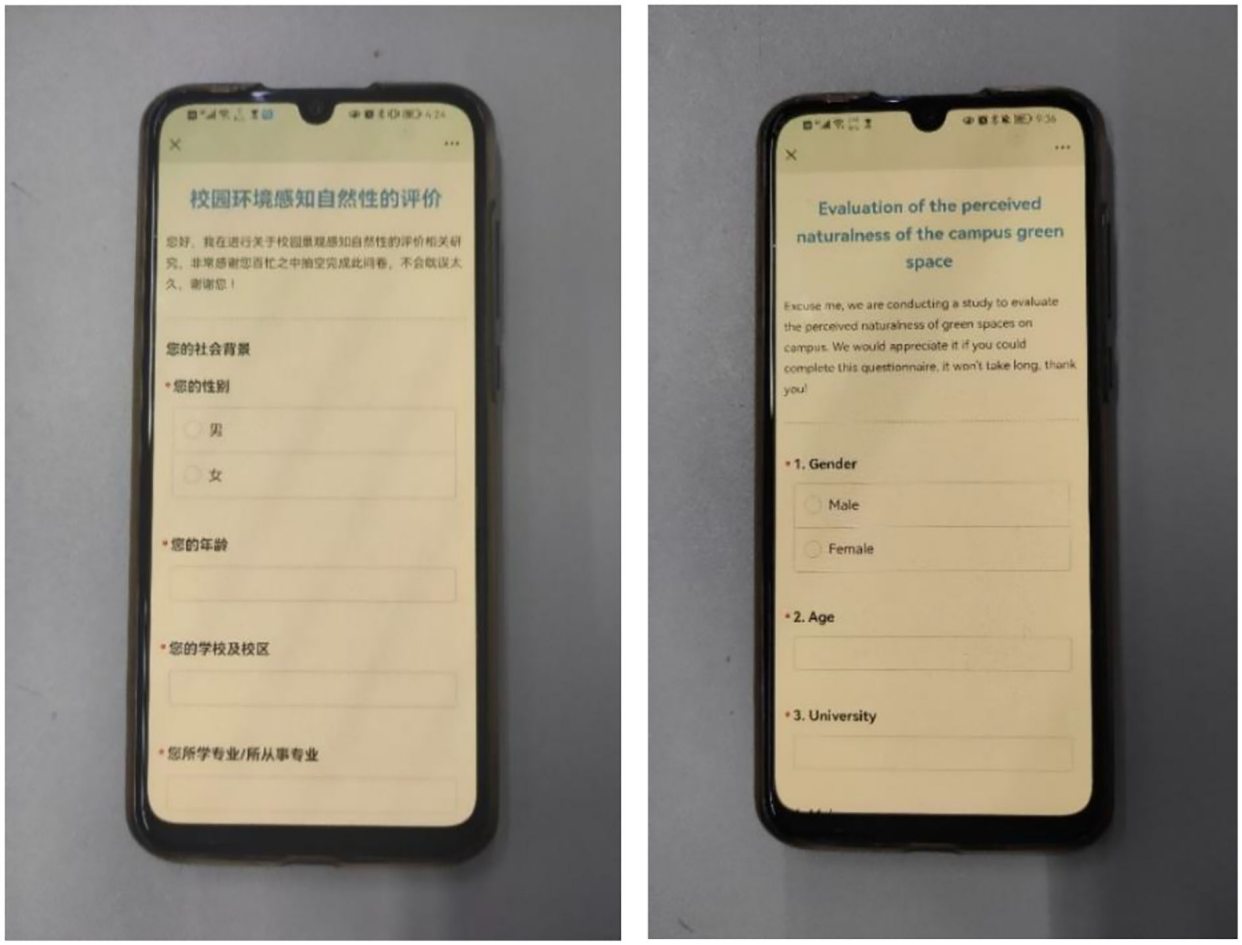
Questionnaire form sample in Chinese and English.

A questionnaire was developed for the survey with five parts. The first part is regard to collecting information on students' socio-demographic characteristics, including gender, age, education level, and monthly living expense. The second part is used to acquire students' perceived naturalness in the form of a self-rated naturalness scale (SRNS) (17). The third part applies the Positive Affect Scale from the Positive and Negative Affect Scale (PANAS) (in Chinese), as a snippet of the full scale, for obtaining students' positive emotions when exposed to the CGSs (60). The fourth part directs at evaluating students' place attachment in their university based on the Place Attachment Scale (PAS) (45). The final section measures students' landscape preference based on the environmental preference matrix (49). Except for the first part, all questions are answered by respondents using a Likert-type scale from 1 to 5 (1-strongly disagree, 2-somewhat disagree, 3-neither disagree nor agree, 4-somewhat agree, 5-strongly agree). The screenshots of the questionnaire forms are shown in Figure 4.

From February to April 2021, students were randomly invited to join the surveys in the two selected universities. All participants voluntarily took part in our surveys, and the collected questionnaire forms are anonymous for privacy issues. Before answering the questionnaire, respondents were informed of the purpose of the questionnaire and asked to recall their emotional states during their routine activities in the CGSs over the past 2 weeks. Finally, of the 539 possible eligible questionnaires, 474 completed questionnaires passed the validity check (UA: 253, UB: 221; effective response rate: 87.94%).

### Structural equation modeling

Structural equation modeling (SEM) is a popular method for data analysis that has been widely used in many fields, such as psychology, sociology, and education. Unlike other modeling approaches, SEM integrates a measurement model and structural model, which can directly respond to the relationship between latent and observed variables, as well as the relationship between latent variables (62).

The equation of the measured model is as follows:


(1) $$\begin{array}{rcl} X & = & \Lambda_{x}\xi \;+\;\delta \end{array}$$

(2) $$\begin{array}{rcl} Y & = & \Lambda_{y}\eta \;+\;\varepsilon \end{array}$$

where *X* denotes the vector consisting of measured variables of the *i*th independent latent variables, *Y* denotes the vector of measured variables of dependent latent variable; Λ_*xi*_ and Λ_*y*_ are matrices of factor loadings for *X* and *Y*, respectively; δ and ε are measurement errors of independent and dependent latent variables, respectively; and ξ is a vector of latent independent variables and η is a vector of latent dependent variables. The variances and covariances among the measurement errors of measured independent variables in *X* are contained in Ω_δ_, and the variances and covariances among the measurement errors for the measured dependent variables are included in Ω_ε_.

The regression equation of the structural model is as follows:


(3) $$\begin{array}{r} \eta \;=\;B\eta \;+\;\Gamma \xi \;+\;\zeta , \end{array}$$

where *B* is the regression coefficient matrix regarding the latent dependent variables; Γ is the regression coefficient matrix relating latent independent variables; and ζ is the vector of the error term in the structural model equation that contains the equation prediction errors. The variances and covariances among the latent independent variables are included in a matrix Φ, and the variances and covariances among latent dependent prediction errors are contained in a matrix Ψ.

To estimate the SEM model, it is assumed in this study that the outcomes were continuous when the random variables follow a normal distribution. Maximum likelihood (ML) was used to figure out the estimation of the model regarding all parameters simultaneously. ML yields an estimate that seeks to maximize the likelihood that the measured data are consistent with the implied model, which is given as (63):


(4) $$\begin{array}{l} F_{ML}\;=\log\left|\Sigma \left(\hat{\Theta }\right)\right|\;+\;tr\left(S\Sigma^{-1}\left(\hat{\Theta }\right)\right)\;-\;\log\left|S\right|\\ \;-\left(p\;+\;q\right) \end{array}$$

where $\sum \left(\hat{\Theta }\right)$ is the matrix that the theoretical model implies composing of the abovementioned matrices of estimated parameters (denoted by $\hat{\Theta }$), including Λ_*xi*_, Λ_*y*_, Ω_δ_, Ω_ε_, *B*, Γ, Φ, and Ψ; *tr* is the trace of the matrix; *S* is the covariance matrix observed in the data; *p* is the number of indicators for exogenous latent variables in the model; and *q* is the number of indicators for the endogenous latent variables in the model. The loadings and coefficients are obtained when the difference between the covariance matrix elements of measured data and the covariance matrix elements implied by the model is minimized.

## Results

### Descriptive statistics

The sociodemographic characteristics of respondents are shown in Table 3. The number of male students who joined the survey at UA was little higher than the number of female students, while the opposite occured at UB. The students in the age group of 22-year-old and below made up the majority of respondents, followed by those aged 23–26 years old. In terms of educational attainment, the largest proportions of respondents are undergraduates, which are 69.17% in UA and 81.00% in UB. Regarding monthly expenditure, students surveyed at both universities mainly spend 1,000-2,000 CNY per month, which account for 65.22% of respondents at UA and 72.40% of respondents at UB. According to the statistical yearbook 2019, the per capita disposable income of urban residents in Changsha is 55,211 CNY (64), while in Harbin, it is 40,007 CNY (65).

**Table 3:** Descriptive statistics on demographic characteristics of the participants.

**Factor**	**Category**	**UA**	**UB**
Sex	Men	136 (53.75%)	99 (44.80%)
	Women	117 (46.25%)	122 (55.20%)
Age (years)	≤ 22	176 (69.57%)	137 (61.99%)
	23-26	63 (24.90%)	82 (37.10%)
	≥ 26	14 (5.53%)	2 (0.91%)
Education	Undergraduate	175 (69.17%)	179 (81.00%)
	Master	73 (28.85%)	41 (18.55%)
	Doctor	5 (1.98%)	1 (0.45%)
Monthly expense (CNY)	≤ 1000	26 (10.28%)	13 (5.88%)
	1000-2000	165 (65.22%)	160 (72.40%)
	≥ 2000	62 (24.50%)	48 (21.72%)

### SEM estimation results

The Tucker–Lewis index (TLI) is widely used to model linear mean and covariance structures. The value of The TLI also ranges between 0 and 1, with results **≥** 0.9, indicating an acceptable fit to the model (66). The comparative fit index (CFI) reflects the difference between the hypothetical model and the independent model. The value of CFI lies between 0 and 1, where a value **≥** 0.9 indicates that the model is acceptable (67). Root Mean Square Error of Approximation (RMSEA) refers to the square root of the asymptotic residual sum of squares. Previous studies showed that an RMSEA of < 0.05 indicates a satisfactory model fit, however, if it is < 0.08, then it indicates an acceptable model fit (68). As shown in Table 4, the model fit indices for both universities meet the desired criteria. The values of TLI and CFI in the estimation of models for the two universities are > 0.9, and the values of RMSEA for the models of both universities are less than the acceptable value of 0.08. These indicate that both the models have good fits.

**Table 4:** Model fit indexes.

	**TLI**	**CFI**	**RMSEA**
UA	0.911	0.918	0.072
UB	0.900	0.910	0.075

As shown in Table 5, the standardized factor loadings between latent variables and all corresponding observed variables are > 0.6. Regarding the structural models, Table 6 shows the estimated results that differ across the two universities. The positive effect of students' perceived naturalness on their positive emotions is significant in two universities. The higher the perceived naturalness that students may have, the more positive emotional states they will reach. Besides the direct influences on positive emotion, in the model of UB, the indirect effect through landscape preference and the joint indirect effect through place attachment and landscape preference are positively significant. However, the indirect effects are not significant when it comes to the model in UA. These are particularly useful for confirming our hypothesis that there are differences in the underlying mechanisms of the relationship between students' perceived naturalness and emotions in different social and environmental contexts. At the same time, these results highlight the necessity of expanding the functionality of the CGSs, which contributes to creating a green environment suitable for restoration and addressing the different psychological needs of students during the COVID-19 pandemic.

**Table 5:** Results of the measurement model.

**Latent variable**	**Indicator**	**UA**		**UB**	
		**Estimate**	***p*-value**	**Estimate**	***p*-Value**
Perceived naturalness	PN01	0.616	0.000	0.661	0.000
	PN02	0.627	0.000	0.707	0.000
	PN03	0.817	0.000	0.896	0.000
	PN04	0.795	0.000	0.904	0.000
	PN05	0.832	0.000	0.861	0.000
Positive emotion	PE01	0.836	0.000	0.816	0.000
	PE02	0.845	0.000	0.795	0.000
	PE03	0.883	0.000	0.838	0.000
	PE04	0.898	0.000	0.872	0.000
	PE05	0.874	0.000	0.858	0.000
	PE06	0.830	0.000	0.908	0.000
	PE07	0.805	0.000	0.795	0.000
	PE08	0.752	0.000	0.753	0.000
	PE09	0.826	0.000	0.772	0.000
Place attachment	PA01	0.802	0.000	0.720	0.000
	PA02	0.718	0.000	0.658	0.000
	PA03	0.771	0.000	0.731	0.000
	PA04	0.634	0.000	0.653	0.000
	PA05	0.830	0.000	0.834	0.000
	PA06	0.807	0.000	0.817	0.000
	PA07	0.878	0.000	0.906	0.000
	PA08	0.806	0.000	0.774	0.000
Landscape preference	LP01	0.843	0.000	0.804	0.000
	LP02	0.845	0.000	0.812	0.000
	LP03	0.844	0.000	0.791	0.000
	LP04	0.789	0.000	0.706	0.000
	LP05	0.791	0.000	0.685	0.000
	LP06	0.708	0.000	0.735	0.000
	LP07	0.645	0.000	0.692	0.000
	LP08	0.843	0.000	0.763	0.000
	LP09	0.780	0.000	0.735	0.000
	LP10	0.689	0.000	0.611	0.000

**Table 6:** Results of the structural model.

**Hypothesis**	**UA**		**UB**	
	**Coefficient**	***p*-Value**	**Coefficient**	***p*-Value**
H1	0.215	0.047	0.247	0.011
H2	0.827	0.000	0.703	0.000
H3	0.166	0.022	0.295	0.000
H4	0.317	0.080	0.151	0.251
H5	0.791	0.000	0.648	0.000
H6	0.235	0.180	0.294	0.045

The direct mediate effect of place attachment is not verified in the models of both universities. This result is inconsistent with recent research, which concluded that place attachment was beneficial to students' positive emotions and mental health (69). One of the possible explanations for this inconformity is that university students' place attachment is likely to link with their past experiences (25). Place attachment is dynamic based on personal experience (70). When people visit an environment, past experiences and memories may be evoked regarding the local landscape elements in their hometowns, which are not aligned with the current environment (24, 71). The irrelevance of the environment to past experiences can decrease people's place attachment (72). Due to the influence of the social context and cultural circumstances on the landscape character of the campus, the CGSs may not meet the environmental needs of students from other regions, which may affect their place attachment to the campus. Another possible explanation is the restriction of the freedom to enjoy the CGSs as a result of the closure of public spaces and the restriction of the distance during the COVID-19 pandemic (73). When students return to the CGSs, their emotional states are getting more positive soon; however, their place attachments are not established immediately. The mediate effects of landscape preference and place attachment have been verified in UB, which indicates that the relationship between students' perceived naturalness of the CGSs, and their positive emotion can be sequentially mediated by place attachment and landscape preference. A campus that is well-designed and meets students' needs for outdoor activities and landscape preferences will have a positive effect on their emotional and psychological recovery (44, 52, 74).

Compared to UB, the joint mediating role of place attachment and landscape preference was not validated at UA. This is mainly due to the absence of a link between students' landscape preferences and positive emotions. People's landscape preferences are heterogeneous and dynamic and can be influenced by temporal, spatial, and personal factors (48). As for UA, the disorder and confusion of the green spaces were caused by the construction on the campus. The campus under construction will generate noise, dust, and visual experiences unrelated to the natural environment (75). Mounting evidence showed that disorder and confusion are determined by the artificial structures in the environment, which can affect students' landscape preferences and positive emotions (4, 52). In addition, the lockdown of universities during the COVID-19 epidemic can also affect students' landscape preferences (76). UA has now gradually reopened with the effective control of the epidemic, but strict social distancing measures are still in place for students (4). Our survey was conducted approximately 5 months after the reopening of UA. This allowed students to spend more time around the campuses and led to increasing familiarity with the CGSs. This familiarity can hinder students' perception of the mystery of the CGSs (4). As a part of students' landscape preference, mystery promises to provide interesting new information through the environment (77). This also implies that the absence of a sense of mystery in the CGSs may have a negative impact on students' landscape preferences; however, it does not influence their positive emotions.

## Discussion and conclusion

This study aimed at understanding the importance of perceived naturalness in university campus greenspaces for improving students' positive emotions, especially after the controlling regulations of the COVID-19 pandemic. The questionnaire-based surveys have been undertaken in two universities in Heilongjiang and Hunan Provinces to collect data on students' positive emotions, perceived naturalness, place attachment, and landscape preference regarding CGSs in different social and environmental contexts.

A comparison of SEM results of the sample groups from the two different universities indicates that perceived naturalness, place attachment, and landscape preference influence positive emotion in different manners. The findings suggest that students' perceived naturalness of the CGSs can directly influence their positive emotions. Meanwhile, students' place attachment and landscape preference can be facilitated by the perceived naturalness of the CGSs, ultimately leading to more positive emotions.

With the improvement of campus environments of Chinese universities in the last two decades, students can experience a more natural and better-designed campus environment than in other urban spaces (78). Nevertheless, more attention is needed to maintain the emotional and psychological well-being of students, especially under the situation of the COVID-19 pandemic. As for the practical implications, this study provides psychological insights into the administration and planning of the university campus. Natural elements, such as plants, lawns, and water bodies, which are expected to make the CGSs more responsive to the needs of students in various social and environmental contexts, are essential for the emotional recovery of students during the COVID-19 pandemic. Campus administrators and planners can take further steps to foster a sense of identity, such as increasing the connectivity, safety, and fun of the CGSs, based on students' common needs during the COVID-19 pandemic. Diverse management and planning measures should be adopted for the CGSs in different periods of the lockdown. Moreover, those students who have severe mental health problems due to the COVID-19 pandemic may have higher rates of infection and mortality (79). In addition to the emotional restoration of the CGSs, there is a need to address the root causes of students' psychological problems through vaccinations and other methods (80). This study effectively establishes the causal relationships between the study variables through students' subjective perceptions, but this approach is prone to perception bias and recall bias. For example, this study only investigated the results of naturalness through perception and evaluation, thus lacking objective indicators of the CGSs for students' perceived naturalness. Technically, the results emerging from field surveys and simultaneous measurements could provide more useful information on how people perceive the naturalness and what environmental variables affect place attachments, as well as the direct effects of environmental variables on students' emotions. As subjective attributes, perceived naturalness, place attachment, and landscape preference all change in response to environmental information and personal states, which may involve individual core values and cognitive transfer processes (81). Therefore, future research is required to employ more complex conceptual frameworks, such as the mind sponge mechanism, to investigate the relationship between environmental variables and subjective perceptions. In this study, data were collected from two universities because of the limitation of human resources and budget. Future studies could extend the number of study locations to avoid sampling bias.

## Data availability statement

The raw data supporting the conclusions of this article will be made available by the authors, without undue reservation.

## Ethics statement

The studies involving human participants were reviewed and approved by Academic Ethics Committee of School of Architecture and Art, Central South University. The patients/participants provided their written informed consent to participate in this study.

## Author contributions

SL, YJ, JL, and ZL carried out investigation and made data curation. SL, YJ, JL, and YP made formal analysis. SL and YJ wrote the original draft preparation. YP, WL, and TF reviewed, revised, and edited the manuscript. All authors contributed to the article and approved the submitted version.

## Conflict of interest

The authors declare that the research was conducted in the absence of any commercial or financial relationships that could be construed as a potential conflict of interest.

## Publisher's note

All claims expressed in this article are solely those of the authors and do not necessarily represent those of their affiliated organizations, or those of the publisher, the editors and the reviewers. Any product that may be evaluated in this article, or claim that may be made by its manufacturer, is not guaranteed or endorsed by the publisher.
